# What are the current anti-COVID-19 drugs? From traditional to smart molecular mechanisms

**DOI:** 10.1186/s12985-023-02210-z

**Published:** 2023-10-24

**Authors:** Sawsan Aboul-Fotouh, Ahmed Nageh Mahmoud, Esraa M. Elnahas, Mohamed Z. Habib, Sahar M. Abdelraouf

**Affiliations:** 1 Department of Clinical Pharmacology, Faculty of Medicine, Ain Shams University, Cairo, Egypt; 2 Clinical Pharmacology Unit, Faculty of Medicine, Ain Shams University, Cairo, Egypt; 3 Department of Biochemistry, Faculty of Pharmacy, Misr International University, Cairo, Egypt

**Keywords:** COVID-19, SARS-CoV-2, Pharmacotherapy

## Abstract

**Background:**

Coronavirus disease 19 (COVID-19) is the disease caused by SARS-CoV-2, a highly infectious member of the coronavirus family, which emerged in December 2019 in “Wuhan, China”. It induces respiratory illness ranging from mild symptoms to severe disease. It was declared a “pandemic” by the World Health Organization (WHO) in March 2020. Since then, a vast number of clinical and experimental studies have been conducted to identify effective approaches for its prevention and treatment.

**Main body:**

The pathophysiology of COVID-19 represents an unprecedented challenge; it triggers a strong immune response, which may be exacerbated by “a cytokine storm syndrome”. It also induces thrombogenesis and may trigger multi-organ injury. Therefore, different drug classes have been proposed for its treatment and prevention, such as antivirals, anti-SARS-CoV-2 antibody agents (monoclonal antibodies, convalescent plasma, and immunoglobulins), anti-inflammatory drugs, immunomodulators, and anticoagulant drugs. To the best of our knowledge, this review is the first to present, discuss, and summarize the current knowledge about the different drug classes used for the treatment of COVID-19, with special emphasis on their targets, mechanisms of action, and important adverse effects and drug interactions. Additionally, we spotlight the latest “October 2023” important guidelines (NIH, IDSA, and NICE) and FDA approval or authorization regarding the use of these agents in the management of COVID-19.

**Conclusion:**

Despite the wide array of therapeutic strategies introduced for the treatment of COVID-19, one of the most prominent therapeutic challenges is SARS-CoV-2 mutations and emerging new variants and subvariants. Currently, the anti-COVID-19 drug pipeline is continuously affording novel treatments to face this growing challenge.

**Supplementary Information:**

The online version contains supplementary material available at 10.1186/s12985-023-02210-z.

## Background

Coronaviruses are a family of viruses that primarily infect animals, including camels, cattle, cats, and bats, but may also infect humans, causing symptoms ranging from common cold-like symptoms to severe respiratory and systemic symptoms [1]. Over the past 20 years, two human outbreaks were caused by coronaviruses: the severe acute respiratory syndrome coronavirus (SARS-CoV-1) in 2002 and the Middle East respiratory syndrome coronavirus (MERS-CoV) in 2012 [2]. SARS-CoV-2 is a recent family member that emerged in “Wuhan, China” as an outbreak of “viral pneumonia” [3]. The World Health Organization (WHO) first reported it on December 31, 2019 (so-called coronavirus disease 19, or COVID-19), then declared it a “pandemic” on March 11, 2020 [4].

According to the WHO weekly epidemiological update, there have been more than 770 million confirmed cases of COVID-19, including more than 6 million deaths as of September 24, 2023. The WHO estimates that about 80% of COVID-19 cases recover without the need for hospital treatment, 15% become seriously ill and require oxygen (O_2_), and about 5% of cases require intensive care [5]. In light of the above, the number of COVID-19 reported cases and the mortality rate have been vastly rising around the world.

## SARS-CoV-2 variants

Viruses naturally mutate over time, resulting in new versions or variants. According to the Centers for Disease Control and Prevention (CDC), there are multiple COVID-19 Variants Being Monitored (VBM), which refers to these variants that do not pose a significant risk to public health. Among these variants is the Alpha (B.1.1.7) variant, which was first reported in May 2020 in the UK. It spreads more easily and can induce more severe illness than previous versions. The Beta (B.1.351) variant was first reported in South Africa in August 2020; it spreads more easily than older strains and has numerous sub-lineages. The Gamma (P.1) variant, which originated in Brazil in November 2020, may be able to re-infect people who have had COVID-19 [6, 7]. The Delta variant (B.1.617.2) was first detected in India in late 2020. This variant is thought to be responsible for India's deadly second wave of the pandemic in February 2021 [8].

According to the CDC, a variant of concern (VOC) is defined as “a variant for which there is evidence of an increase in transmissibility, more severe disease (for example, increased hospitalizations or deaths), significant reduction in neutralization by antibodies generated during previous infection or vaccination, reduced effectiveness of treatments or vaccines, or diagnostic detection failures” [7]. Now, only the Omicron (B.1.1.529) variant is classified as a VOC [7]. This variant was first detected in South Africa in November 2021 [9]. It has many mutations (> 30 substitutions, deletions, or insertions) in the spike protein, and it has raised concerns that this variant could escape from protection conferred by vaccines and therapeutic monoclonal antibodies [7, 8].

The Omicron variant is 91% less fatal than the Delta variant, with 51% less risk of hospital admission [10]. This may be attributed to its low ability to penetrate deep lung tissue [11]. The main challenge with the Omicron variant is its rapid multiplication in the bronchi (70 times faster than the Delta variant), the high rate of spread, and the ability to escape double-dose vaccination, which led to an increase in the number of patients requiring hospital care [12, 13]. Interestingly, double-dose vaccination could offer 30–40% protection against infection and 70% protection against hospitalization resulting from Omicron infection, but a recent third dose could boost effectiveness against infection to around 75% and 88% for severe disease [14].

There is an increasing number of Omicron subvariants; these variants may differ in the rate of transmission, the severity of disease, and the resistance to anti-SARS-CoV2 monoclonal antibodies. On September 30, 2023, the subvariant “EG.5”, which is called “Eris”, was the most prevalent in the USA (29.4%), according to the CDC COVID data tracker [15].

## SARS-CoV-2 structure and replication cycle

SARS-CoV-2 shares about 79% of its genetic sequence with SARS-CoV-1 and 50% with MERS-CoV [16]. It consists of four main structural proteins: the S (spike), membrane (M), envelope (E), and N (nucleocapsid) proteins. The N protein contains the RNA genome, while the S, E, and M proteins form the envelope of the virus [17]. S proteins are type 1 fusion glycoproteins that are divided into two types (S1 and S2). S1 represents the “surface-exposed” part, which contains the receptor binding domain, while S2 represents the transmembrane part, which is responsible for the fusion of viral and cellular membranes [18].

**Fig. 1:**
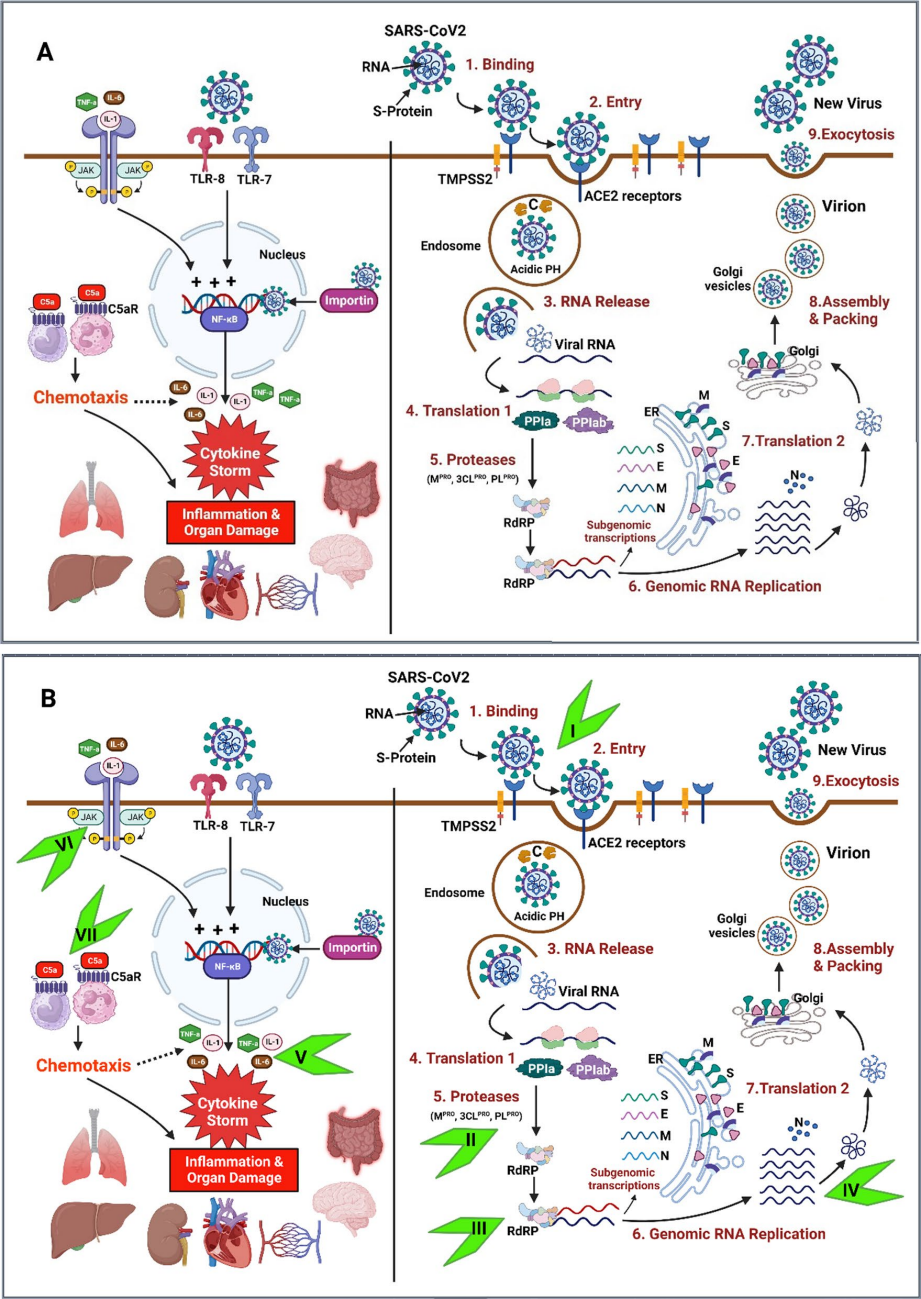
**A** SARS-CoV-2 replication cycle (on the right half) and pathophysiology of COVID-19 (on the left side). **B** Current anti-COVID-19 drug targets and sites of action. **I:** Drugs that target the S-protein, inhibiting S-protein-ACE2 interaction and viral entry to the host cell, e.g., SARS-CoV-2 monoclonal antibodies, convalescent plasma neutralizing antibodies. **II:** Protease inhibitors, e.g., Paxlovid (nirmatrelvir/ritonavir). **III:** RNA-dependent RNA polymerase (RdRp) inhibitor, e.g., remdesivir. **IV:** Molnupiravir, which is incorporated into viral RNA strands, leading to ‘error catastrophe’ during viral replication. **V:** Anti-inflammatory and immunosuppressants, e.g., corticosteroids, IL-6 inhibitors, and IL-1 inhibitors. **VI:** JAK Inhibitors, e.g., baricitinib and tofacitinib. **VII:** Complement component “C5a” inhibitors, e.g., Vilobelimab

As depicted in Fig. 1A, COVID-19 infection starts when the virus binds to human cell surface receptors [19]. The virus was reported to show affinity to human angiotensin-converting enzyme 2 (ACE2), with more affinity compared to SARS-CoV-1 and MERS-CoV [20]. Indeed, the S-protein-ACE2 interaction represents the principal mechanism of SARS-CoV-2-mediated host cell invasion [21, 22].

After engaging ACE-2, the S-protein is exposed to cleavage by furring into S1-S2 subunits, which is a prerequisite for S2′ site cleavage either by the transmembrane protease serine 2 (TMPRSS2) on the cell surface or by cathepsins in the endosomes following ACE2-mediated endocytosis [23]. Cathepsins require an acidic environment for their optimal activity, which promotes the release of the virus’s genome into the host cells [23]. This is followed by the translation of the viral genome into the viral replicase polyproteins (PP1a and PP1ab), which are cleaved into functional or nonstructural proteins (nsp1-16) by the viral proteases, the main protease (Mpro), and the papain‐like protease (PLpro) [24, 25].

Subsequently, the SARS-CoV-2 genome replicates using the RNA-dependent RNA polymerase (RdRp) enzyme with instantaneous transcription of structural protein-coding messenger RNAs, which are then translated and assembled into new viral structural proteins. Afterward, the packing of viral genomes by viral structural proteins occurs, producing viral particles or virions surrounded by Golgi vesicles. Lastly, the mature virions are released by exocytosis [16, 26].

The primary target of SARS-CoV-2 is the respiratory and gastrointestinal tract; this could be a result of the “cell tropism” for the nasal epithelial cells, the alveolar macrophages, and the GIT enterocytes [26]. Due to its abundance in the bronchial fluids, sputum, and saliva, the main route of viral transmission is through the inhalation of aerosols, droplets of saliva, or nasal discharge of infected individuals [27].

## Pathophysiology and clinical picture of COVID-19

After entry into the host cell, the virus is recognized by Toll-Like Receptors 7 and 8 (TLR7/8), cytosolic RNA sensors RIG-I/MDA-5, and the inflammasome sensor NLR family pyrin domain-containing-3 (NLRP3), with subsequent activation of NF-кB and IRF3/7 and production of pro-inflammatory cytokines (e.g., IL-1β, IL-6, and TNF-α) and type I IFNs [28]. Cytokines released by infected cells serve to modulate the adaptive immune response by enhancing the recruitment and activation of macrophages, B, and T cells to facilitate the elimination of the virus. However, an unbalanced immune response can cause a massive release of pro-inflammatory cytokines, leading to a “cytokine storm syndrome” responsible for organ damage, acute respiratory distress syndrome (ARDS), and respiratory failure, which represent the main cause of death in COVID-19 patients [29].

The primary site of SARS-CoV-2 morbidity is the respiratory tract. Nevertheless, extrapulmonary manifestations are also common, affecting the heart, liver, kidney, brain, intestine, pancreas, testes, ovaries, breast, uterus, and placenta. This might be attributed to the high expression of ACE2 in these tissues [30, 31].

Another pivotal mechanism underlying the pathophysiology of multi-organ injury secondary to SARS-CoV-2 infection includes direct viral endothelial injury, which may induce inappropriate thrombin formation with inhibition of fibrinolysis and activation of complement pathways, predisposing microthrombi deposition, and microvascular dysfunction [30]. Moreover, the dysregulation of the renin–angiotensin–aldosterone system (RAAS) may trigger vasoconstriction, inflammation, and tissue injury [32].

Multiple clinical studies have detected antiphospholipid antibodies (APLs) in individuals infected with SARS-CoV-2. APLs are recognized to enhance platelet activation and pro-coagulants synthesis, thus encouraging thrombogenesis in COVID-19 patients, and this could be explained by two potential mechanisms: molecular mimicry and neoepitope formation [33].

**Fig. 2:**
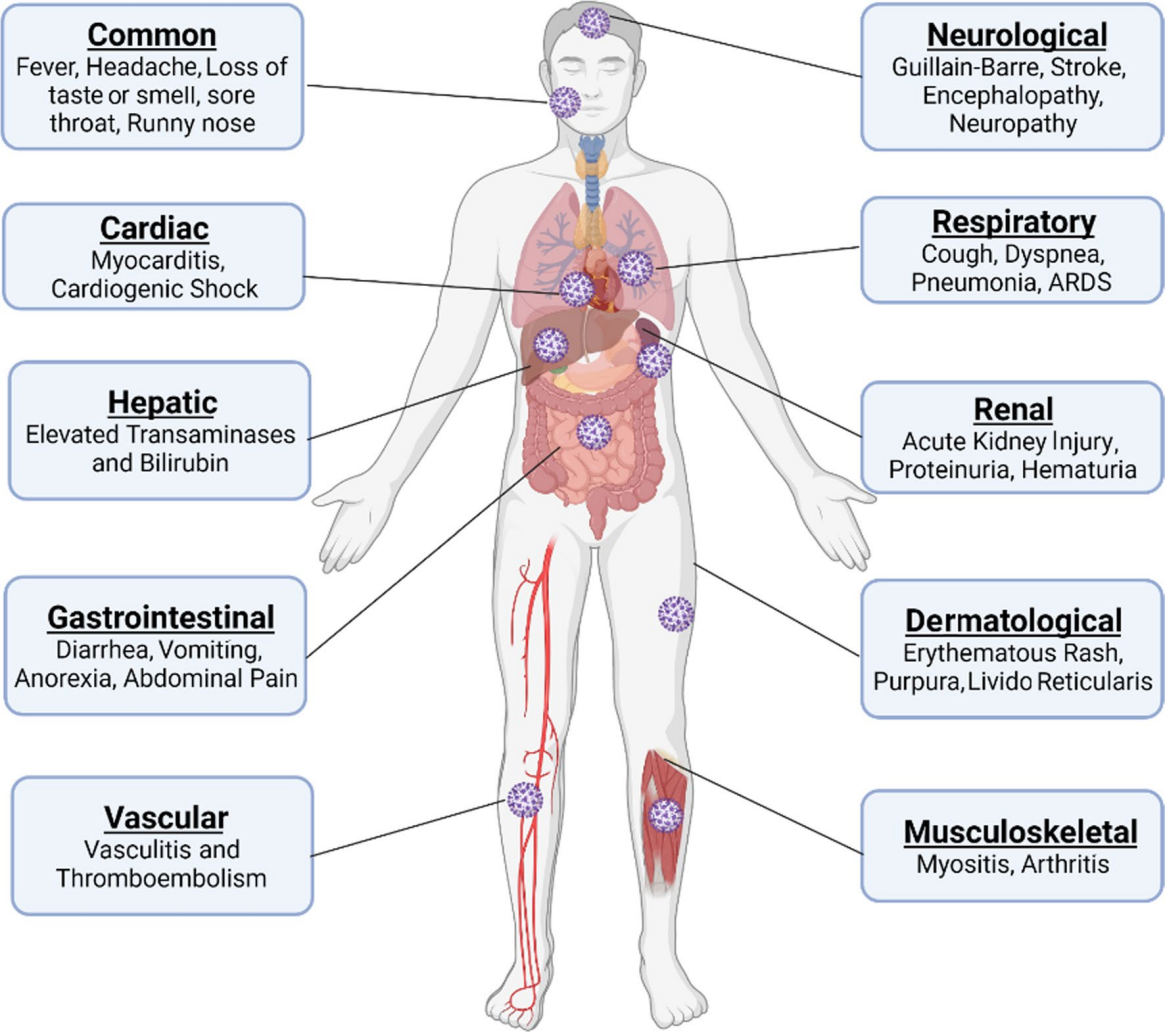
Clinical picture of COVID-19

As illustrated in Fig. 2, the clinical presentation of COVID-19 includes pulmonary and extrapulmonary manifestations. According to the WHO, fever, dry cough, and fatigue are the most commonly experienced symptoms of COVID-19. Other less common symptoms include nasal congestion, conjunctivitis, nausea and/or vomiting, diarrhea, loss of smell, sore throat, headache, muscle or joint pain, dizziness, and skin rash. However, with more severe disease, patients may suffer from shortness of breath, confusion, chest pain, and fever [34].

Other manifestations reported include hematological findings like lymphopenia, which is a quite common lab finding reported in 67–90% of patients with COVID-19, coagulopathy, and increased D-dimer levels [35]. Cardiovascular manifestations include acute coronary syndrome and myocardial injury, with the elevation of cardiac markers up to cardiogenic shock [36]. Acute kidney injury, hematuria, proteinuria, hyperkalemia, and acidosis were also reported with COVID-19. Liver enzymes may be elevated, and elevated bilirubin on admission is linked to severity and progression. Multiple neurological manifestations have been reported, ranging from anosmia to acute stroke and Guillain-Barré syndrome [37].

The main causes of death are respiratory failure, ARDS, sepsis, thromboembolism, and multi-organ failure (heart, liver, and kidney). Older patients (> 60 years), smokers, and patients with serious pre-existing diseases are at increased risk of developing ARDS and death [33].

## Drug therapy for COVID-19

Different drug classes have emerged during the COVID-19 pandemic, and multiple drugs (with other indications) have been “repurposed” and showed substantial efficacy against the virus. These drugs can be classified as antivirals, anti-SARS-CoV-2 antibody agents (monoclonal antibodies, convalescent plasma, and immunoglobulins), anti-inflammatory, and immunomodulators (Fig. 1B).

In this review, we discuss and summarize the different drug classes used for the treatment of COVID-19, with special emphasis on their targets, mechanisms of action, adverse effects, and drug interactions (summarized in Table 1). Additionally, we spotlight the latest important guideline recommendations “October 2023” of NIH [38], IDSA [39], and NICE [40] and FDA approval or authorization regarding the use of these drugs in the management of COVID-19 (summarized in Additional file 1: Table 1).

**Table 1:** Current approved/authorized COVID-19 drugs: mechanisms of action in COVID-19, other uses, adverse effects, and precautions

Drug	Indications in Covid 19	Mechanism of action in COVID-19	Other uses	Adverse Effects and Precautions
*I. Antiviral Drugs*				
Remdesivir	FDA-approved for the treatment of adults and pediatric patients with mild-to-moderate COVID-19 at high risk of disease progression	Adenosine analog inhibiting viral RNA-dependent RNA polymerase → inhibition of viral replication	Ebola virus	↑ Liver transaminases
	Recommended by NIH, IDSA, and NICE guidelines	Effective against the Omicron variant	SARS-CoV-1	↑PT, Nausea
				Hypersensitivity
				Not recommended for patients with eGFR < 30 mL/min
Ritonavir/Nirmatrelvir (Paxlovid)	FDA-approved (May 2023) for the treatment of adults with mild-to-moderate COVID-19 at high risk of disease progression	A protease inhibitor		Diarrhea, impaired taste, hypertension, and myalgia
	Recommended by NIH, IDSA, and NICE guidelines			Not recommended in patients with severe renal or hepatic impairment
				Used cautiously in patients with liver diseases
				May induce HIV-1 drug resistance
Molnupiravir	FDA-authorized (December 2021) for the treatment of certain adults with mild-to-moderate COVID-19 at high risk of disease progression as an alternative to Paxlovid and remdesivir	Incorporates into viral RNA strands → ‘error catastrophe’ during viral replication		Diarrhea, nausea, and dizziness
	Recommended by NIH, IDSA, and NICE guidelines	Effective against the Omicron variant		Not recommended in pregnancy and lactation
				Not authorized for patients ≤ 18 years with COVID-19 due to bone and cartilage growth affection
*II. Anti-SARS-CoV2 antibody agents*				
Sotrovimab “Anti-SARS-CoV-2 monoclonal antibodies”	Sotrovimab is recommended by the NICE for the treatment of COVID-19 as an alternative to Paxlovid in patients with increased risk for disease progression only if they do not need supplemental O_2_	Single mAb that binds to and blocks S-protein of SARS-CoV-2		Hypersensitivity, anaphylaxis (infusion-related reactions)
		Effective against Omicron variant (B.1) but not against subvariants B.2		
COVID-19 Convalescence Plasma (CCP)	FDA-authorized (August 2020) for the treatment of COVID-19 in patients with immunosuppressive disease or receiving immunosuppressive treatment	Antiviral effects of neutralizing antibodies (NAbs) IgM and IgG against S-protein	CCP is widely used in outbreaks and epidemics until reaching a definitive treatment	Mild allergic reaction, nausea, skin erythema, and fever
	Suggested by the IDSA in ambulatory patients with mild-to-moderate COVID-19 at high risk of disease progression who have no other treatment options	NAbs → ↓autoantibodies, cytokine storm, Th1/Th17 ratio, complement		Transmission of infection as HIV, HBV, or HCV
		NAbs → ↑ IL-10		
		Regulate coagulation and ↓Clotting		
*III. Anti-inflammatory and immunomodulatory drugs*				
Corticosteroids	Life-saving—recommended by the WHO for patients with severe or critical COVID-19	To suppress both acute respiratory distress syndrome (ARDS) and systemic inflammation	Wide range of uses including autoimmune diseases, inflammatory diseases, and organ transplantation	Hyperglycemia, neuropsychiatric symptoms, secondary infections with ↑risk of opportunistic fungal infections (e.g., mucormycosis, aspergillosis), and reactivation of latent infections (e.g., HBV, herpesvirus infections, and TB)
	Recommended by NIH, IDSA, and NICE guidelines			
Interleukin-6 inhibitors	Tocilizumab is FDA-approved (December 2022) for the treatment of hospitalized adults with COVID-19 who are receiving systemic corticosteroids and require supplemental O_2_, mechanical ventilation, or ECMO	↓ Cytokine storm	Rheumatoid arthritis and giant cell arteritis	Runny nose, sore throat, sinus infection, headache
Tocilizumab	Recommended by NIH, IDSA, and NICE guidelines			High blood pressure
Sarilumab				Injection site reactions
				GIT perforations
Interleukin-1 inhibitors	Anakinra is FDA-authorized (November 2022) for the treatment of hospitalized adults with COVID-19 with pneumonia requiring supplemental O_2_ who are at risk of disease progression and likely to have an elevated plasma soluble urokinase plasminogen activator receptor	↓ Cytokine storm	Rheumatoid Arthritis	Headache, nausea, vomiting, and liver enzyme elevations
Anakinra			Cryopyrin-associated periodic syndromes (CAPS)	
Canakinumab				
JAK Inhibitors	Baricitinib is FDA-approved (May 2022) for the treatment of hospitalized adults with COVID-19 requiring supplemental O_2_, mechanical ventilation, or ECMO	Inhibits JAK1/JAK2 → inhibition of the inflammatory cascade	Rheumatoid arthritis	Hypersensitivity reactions
Baricitinib	Recommended by NIH, IDSA, and NICE guidelines	Inhibits IL-6-induced STAT3 phosphorylation	Psoriatic arthritis	Infections: respiratory and urinary tract infections, reactivation of herps
Tofacitinib		Inhibition of viral entry into the host cell	Ulcerative colitis	Myelosuppression, thrombosis, elevation of liver enzymes
				Cardiac-related events (MI and stroke)
				Baricitinib needs dose adjustment in renal patients
Vilobelimab	FDA-authorized (April 2023) for the treatment of hospitalized adults with COVID-19 when initiated within 48 h of receiving invasive mechanical ventilation or ECMO	A monoclonal antibody that binds to the soluble form of the complement component “C5a” → inhibits the inflammatory response → ↓ Cytokine storm	-	Pneumonia, sepsis
				Infections: herpes simplex, enterococcal infection, bronchopulmonary aspergillosis
				Delirium
				Pulmonary embolism, DVT
				Hypertension
				Elevated liver enzymes
				Thrombocytopenia
				Rash

FDA, U.S. Food and Drug Administration; NIH, National Institutes of Health; IDSA, Infectious Diseases Society of America; NICE, National Institute of Health and Care Excellence; eGFR, estimated Glomerular Filtration Rate; O_2_, Oxygen; IL-10, Interleukin-10; HIV, Human Immunodeficiency Virus; HBV, Hepatitis B Virus; HCV, Hepatitis C Virus; TB, Tuberculosis; ECMO, Extracorporeal Membrane Oxygenation; GIT, Gastrointestinal Tract; MI, Myocardial Infarction; DVT, Deep Venous Thrombosis

### Anti-viral drugs

This class of drugs works by interfering with the SARS-CoV-2 replication cycle to reduce the viral load and its subsequent pathological effects. Antiviral drugs inhibit the entry of the virus through the ACE2 receptor and/or TMPRSS2, the viral membrane fusion and endocytosis, or the viral proteases and RdRp. This class of drugs has a vital role in preventing COVID-19 illness progression because viral replication is more active during early infection [41].

#### Currently approved COVID-19 antiviral drugs

##### Remdesivir

Remdesivir is the first FDA-approved antiviral drug against COVID-19. It is a nucleotide prodrug, and its active metabolite, which is an adenosine analog, can bind to viral RdRp and inhibit viral replication through premature termination of RNA transcription [42].

In 2020, the WHO recommended against the use of remdesivir in COVID-19 patients regardless of disease severity due to the lack of evidence at that time that remdesivir could improve survival or any other clinical outcomes. In April 2022, following the emergence of new data from clinical trials, the WHO updated its recommendations and suggested the use of remdesivir in mild or moderate COVID-19 patients who are at high risk of hospitalization [43, 44].

The FDA and the latest guideline (NIH, IDSA, and NICE) versions recommend the use of remdesivir in hospitalized and non-hospitalized adult and pediatric (aging ≥ 28 days and weighing ≥ 3 kg) patients with “mild to moderate COVID-19” to reduce the risk of disease progression. Additionally, NIH recommends the co-administration of remdesivir with dexamethasone for hospitalized COVID-19 patients requiring O_2_ supplementation [38–40].

Remdesivir is administered by intravenous (IV) route at a dose of 200 mg infused over 30–120 min on day 1 (loading dose) followed by 100 mg/day. For pediatric patients (less than 40 kg), the loading dose is 5 mg/kg on day one, followed by 2.5 mg/kg/day. The most common adverse effect of remdesivir is nausea. It may also elevate liver transaminases and prothrombin time and induce hypersensitivity reactions. Chloroquine and hydroxychloroquine reduce remdesivir antiviral effectiveness; therefore, their coadministration is not recommended. Remdesivir dose should be adjusted in patients with renal insufficiency. Its use is not recommended in patients with an estimated glomerular filtration rate (eGFR) < 30 mL/min. Remdesivir is well tolerated during pregnancy, with a low rate of serious adverse effects [45].

##### Paxlovid

Paxlovid is the first FDA-approved oral antiviral drug against COVID-19 [46]. It is a combination of nirmatrelvir, which inhibits the main protease (Mpro) of SARS-CoV-2, and ritonavir, the inhibitor of cytochrome P450-3A4, thus slowing down nirmatrelvir metabolism. This combination allows a longer half-life of nirmatrelvir, allowing a 12-h dosing interval. It is the first oral anti-viral drug approved for COVID-19 [46].

The FDA and the latest guideline (NIH, IDSA, and NICE) versions recommend the use of Paxlovid in non-hospitalized adult and pediatric (≥ 12 years and ≥ 40 kg) patients with “mild to moderate COVID-19” to reduce the risk of disease progression [38–40, 46].

Paxlovid side effects include diarrhea, impaired taste, hypertension, and myalgia. It is not recommended for patients with severe renal or hepatic impairment. It should be used cautiously in patients with liver diseases, abnormal liver enzymes, or hepatitis [39]. Using Paxlovid in people with uncontrolled or undiagnosed HIV-1 infection may induce HIV-1 drug resistance [47].

Paxlovid is contraindicated in patients with a history of clinically significant hypersensitivity reactions. As it is a CYP-3A4 inhibitor, it is contraindicated in patients receiving drugs metabolized by CYP-3A4, like alfuzosin, colchicine, propafenone, amiodarone, ergotamine, statins, sildenafil, midazolam, and triazolam [47]. The dose of Paxlovid should be adjusted in patients with eGFR ≤ 60 mL/min. Paxlovid is not recommended for patients with eGFR < 30 mL/min [39].

##### Molnupiravir

Molnupiravir is another oral antiviral drug that targets viral replication. It is a prodrug that is converted to β-D-N4-hydroxycytidine (NHC), which is incorporated into viral RNA strands mimicking nucleoside cytidine or uridine and leading to ‘error catastr5phe’ during viral replication [48].

The FDA and the guidelines of NIH, IDSA, and NICE recommend the use of molnupiravir in non-hospitalized adult patients with “mild to moderate COVID-19” to reduce the risk of disease progression “only when Paxlovid or remdesivir cannot be used” [38–40, 49]. The dose of molnupiravir is 800 mg orally every 12 h for 5 days, starting within 5 days of symptom onset [38].

The most common side effects of molnupiravir are nausea, diarrhea, and dizziness. Neither drug interactions nor contraindications were reported, as the available data is limited. However, it is not authorized for patients ≤ 18 years with COVID-19 due to bone and cartilage growth affection and is not recommended in pregnant or lactating females. Additionally, molnupiravir is not FDA-authorized for pre- or post- exposure prophylaxis of COVID-19. Due to its lack of clinical benefit, molnupiravir is not authorized for the treatment of hospitalized COVID-19 patients [39].

#### Currently used, not approved COVID-19 antiviral drugs

##### Nitazoxanide

This antiprotozoal agent was first approved by the FDA for the treatment of Giardia duodenalis and cryptosporidium parvum in adults and children > 1 year [50]. It is a prodrug that is actively metabolized to its active form, tizoxanide, which interferes with the pyruvate: ferredoxin oxidoreductase (PFOR) enzyme-dependent electron transfer reaction necessary for the anaerobic metabolism in anaerobic organisms. It has shown in vitro anti-viral activity against multiple viruses like the influenza virus, Rotavirus, Norovirus, hepatitis B and C, Ebola virus, MERS-CoV, and COVID-19 [50–52].

Nitazoxanide inhibits host enzymes, which impairs the post-translational processing of viral proteins. It also has an inhibitory effect on pro-inflammatory cytokine production. The NIH “recommends against the use of nitazoxanide for the treatment of COVID-19, except in a clinical trial” [38]. Interestingly, multiple clinical trials highlighted the role of nitazoxanide in reducing the risk of COVID-19 progression, decreasing the median time for clinical recovery, and reducing the SARS CoV-2 viral load [53–55]. Indeed, the nitazoxanide/azithromycin combination has been suggested as a new protocol for early management of COVID-19 [56].

Nitazoxanide is a well-tolerated drug; however, it is associated with some side effects, including nausea, vomiting, and abdominal pain, and urine and ocular discoloration (rare). Nitazoxanide is highly bound to plasma protein (> 99.9%); therefore, drug-drug interactions may occur when nitazoxanide is concurrently administered with other highly plasma protein-bound drugs due to the competition for plasma protein binding sites [57].

##### Azithromycin

Azithromycin is a macrolide broad-spectrum antibiotic that mediates its anti-bacterial effects via protein synthesis inhibition [58]. It has shown anti-viral activity against multiple viruses, including Ebola and Zika viruses [57–60]. However, its anti-viral mechanism is not yet clearly identified [60]. Moreover, macrolides have been shown to exhibit an anti-inflammatory effect [61, 62].

As regards COVID-19, which is characterized by exacerbated inflammation, azithromycin was shown to suppress pro-inflammatory cytokine production. It also inhibits T cells by inhibiting calcineurin signaling, mammalian target of rapamycin activity (mTOR), and NFκB activation [63].

The NIH, the IDSA, and the NICE guidelines do not recommend azithromycin for the treatment of COVID-19 in the absence of other indications [38–40].

Adverse effects include allergy, hepatotoxicity, QT prolongation, ventricular tachycardia, and gastrointestinal upset, which need to be taken into consideration, especially in the outpatient setting where frequent ECG monitoring may not be possible [39]. Although azithromycin has a minimal risk for cytochrome P450 interactions, serious drug interactions with other antivirals or drugs that induce QT interval prolongation should be considered [39].

#### Obsolete COVID-19 antiviral drugs

##### Favipiravir

It is a RdRp inhibitor like remdesivir. It is a prodrug purine analog, and its activated phosphor-ribosylated form (favipiravir-RTP) inhibits viral RNA polymerase activity and genome replication [64]. Favipiravir was approved in 2014 for the treatment of influenza viruses in Japan [64, 65]. Due to the urgency of the situation of COVID-19, favipiravir was repurposed and used (off-label) for the treatment of mild non-hospitalized cases of COVID-19 [66, 67]. However, the NIH, the IDSA, and the NICE guidelines do not recommend or approve using favipiravir for COVID-19 treatment.

In vitro studies revealed that favipiravir may be effective against SARS-CoV-2 [66]. Nevertheless, there is controversy regarding its effectiveness against COVID-19 in clinical trials [67, 68]. A meta-analysis showed that favipiravir reduced the mortality rate by 30%, but this finding was not statistically significant. Moreover, favipiravir treatment induced a significant clinical improvement compared to the control group after 7 days post-hospitalization. On the other hand, after 14 days post-hospitalization, clinical improvement was 10% higher in the favipiravir group, but this finding was also not statistically significant [69]. Clinical evidence supports the safety and tolerability of short-term use of favipiravir [67]. The most reported adverse effect of favipiravir is the elevation of liver transaminases, bilirubin, and uric acid, as well as gastrointestinal disturbances, chest pain, and teratogenicity; therefore, it is contraindicated in pregnancy [70].

##### Lopinavir/ritonavir

Lopinavir/ritonavir is a protease inhibitor approved by the FDA in 2000 for the treatment of HIV [71]. Ritonavir is added as it is a cytochrome P450-3A4 inhibitor to slow lopinavir metabolism. This combination exhibited in-vitro inhibition of SARS-CoV-1 and MERS-CoV replication [72, 73] and reduced ARDS mortality in clinical trials [74].

In the early phase of COVID-19, a triple combination of interferon beta-1b, ribavirin, and lopinavir/ritonavir shortened the duration of hospital stay in patients with mild to moderate COVID-19 in an open-label, randomized, phase II trial [75].

Lopinavir/ritonavir did not show clinical efficacy among non-hospitalized patients with COVID-19 in two RCTs [76, 77]. The NIH and the IDSA strongly recommend against the use of lopinavir/ritonavir for the treatment of COVID-19 in hospitalized or non-hospitalized patients or for post-exposure prophylaxis [38, 39].

The most common reported side effects include nausea, vomiting, diarrhea, abdominal pain, loss of appetite, bloating, metallic taste, paresthesia, itching, prolonged QT interval, and hepatotoxicity, in addition to drug interactions due to its CYP3-A4 inhibiting activity [39].

##### Chloroquine and hydroxychloroquine

Chloroquine and its analog hydroxychloroquine, are used to treat malaria as well as autoimmune diseases like systemic lupus erythematosus and rheumatoid arthritis due to their effect on cytokines like IL-1 and IL-6 [78]. Evidence suggests that these agents may exhibit an effect against multiple viruses, including coronaviruses [79].

They have shown in vitro activity against SARS-CoV-2 within the range of predicted achievable tissue concentrations. This in-vitro effect, the wide use for other diseases, and the common availability of the drug made it a great option for the treatment of COVID-19 [41, 80, 81].

Chloroquine increases the endosomal pH, thus inhibiting the fusion between SARS-CoV-2 and the cell membrane [78]. It also inhibits glycosylation of the ACE2 receptor, which interferes with virus binding [81]. In vitro studies suggest that chloroquine and hydroxychloroquine block the transport of SARS-CoV-2 from endosomes to endolysosomes, thus possibly preventing the release of viral genetic material [82]. Also, hydroxychloroquine inhibits the cytokine storm induced by SARS-CoV-2 via suppressing T-cell activation [83].

However, the NIH and the IDSA guidelines recommend against the use of chloroquine and hydroxychloroquine for the treatment of COVID-19 in hospitalized or non-hospitalized patients due to the lack of clinical benefit among the different RCTs established [38, 39].

Adverse effects of both chloroquine and hydroxychloroquine are nausea, vomiting, dyspepsia, abdominal pain, pruritis, skin rash and discoloration (contraindicated in psoriasis), retinal degeneration and corneal opacities, quinidine-like action with QT prolongation, and hemolytic anemia in G6PD-deficient subjects. In addition to the reported drug interactions, including CYP-2D6 inhibition leading to decreased antiviral activity of remdesivir, therefore, co-administration of these drugs is not recommended [39].

##### Ivermectin

It is an anti-parasitic FDA-approved drug used in diseases like onchocerciasis, head lice, scabies, strongyloids, ascariasis, and filariasis. It is also used in malaria by killing the mosquito, thus preventing the transmission of the infection [84]. Its antiparasitic mechanism is the opening of glutamate-gated and gamma-aminobutyric acid (GABA)-gated chloride channels, leading to an increase in chloride ion conductance, which induces motor paralysis in parasites. However, its mechanism in COVID-19 infection is different; it inhibits the virus binding to the host cell membrane via interfering with ACE-2 receptors and reducing virus/cell fusion [85]. It also inhibits viral nuclear accumulation by blocking the importin α/β protein receptor, which is responsible for the nuclear transport of viral proteins, leading to an efficient antiviral response [86].

Ivermectin was reported to have an anti-inflammatory effect, which was useful in COVID-19 patients [87–89]. Despite having in vitro activity against viruses, e.g., HIV, yellow fever, Zika virus, and dengue fever, no clinical trials have reported clinical significance [90, 91].

In April 2020, the FDA issued a statement concerning the self-administration of ivermectin against COVID-19 and highlighted that those in vitro studies are not sufficient and further trials are needed to confirm the safety and efficacy of ivermectin for its use in COVID-19 patients [92].

The NIH, the NICE, and the IDSA guidelines do not recommend ivermectin for the treatment of COVID-19 except in clinical studies [38–40]. Ivermectin is tolerated; adverse effects of ivermectin include dizziness, pruritis, nausea, or diarrhea [38, 39].

### Anti-SARS-CoV-2 antibody agents

The target of this group is to decrease the viral load in the upper and lower respiratory airways of the infected host, resulting in reduced virus-induced pathology [93, 94]. This group includes anti-SARS-CoV-2 monoclonal antibodies, convalescent plasma, and SARS-CoV-2 specific immunoglobulins.

#### Anti-SARS-CoV-2 monoclonal antibodies (mAbs)

They target the S protein, the main protein used by the virus to attach and fuse to the human cell membrane, thus blocking viral entry into host cells. There are 5 anti**-**SARS-CoV-2 mAbs, including 3 double mAbs, which are bamlanivimab/etesevimab, casirivimab/imdevimab (REGEN-COV), and tixagevimab/cilgavimab (Evusheld), and 2 single mAbs, which are sotrovimab and bebtelovimab [93].

Bamlanivimab (700 mg)/etesevimab (1400 mg) is administered as a single IV injection. They bind to different (but overlapping) epitopes of the S-protein receptor binding domain (RBD). Reported adverse effects of bamlanivimab/etesevimab are nausea, dizziness, pruritis, and hypersensitivity reactions (anaphylaxis and infusion-related reactions) [95].

Casirivimab (600 mg)/imdevimab (600 mg) is administered as a single IV or subcutaneous (SC) injection. They bind to non-overlapping epitopes of the S protein RBD of SARS-CoV-2. Allergic and injection site reactions are the most common adverse effects encountered with casirivimab/imdevimab [96].

Sotrovimab was originally identified in 2003 from SARS-CoV survivors derived from memory B-cells. It is administered at a dose of 500 mg as a single IV infusion. It binds to a conserved epitope on the S-protein RBD of SARS-CoV-2. The reported adverse effects include rash, diarrhea, anaphylaxis, and infusion-related reactions [97].

Tixagevimab co-packaged with cilgavimab (Evusheld) represent other monoclonal antibodies that can bind to non-overlapping epitopes of the S-protein RBD. These drugs were the first FDA-authorized anti-SARS-CoV-2 monoclonal antibodies for the pre-exposure prophylaxis of COVID-19 in adults and pediatric individuals (≥ 12 years). Evusheld is administered as an initial dose of tixagevimab (300 mg) and cilgavimab (300 mg) as 2 separate consecutive intramuscular injections, followed by the repeat dosage of tixagevimab (300 mg) and cilgavimab (300 mg) every 6 months. The most frequently encountered adverse effects of Evusheld were headache, fatigue, cough, hypersensitivity reactions, and anaphylaxis [98].

Between November 2020 and February 2022, multiple anti-SARS-CoV-2 monoclonal antibodie**s** were FDA-authorized for the treatment/prevention of COVID-19 in adults and pediatric patients (≥ 12 years of age) [95–99]. Unfortunately, the extensive mutations of the S protein of the Omicron variant and the subsequent high prevalence of Omicron subvariants resulted in a marked resistance to the action of the therapeutic neutralizing mAbs. Consequently, all clinically authorized therapeutic mAbs targeting the Omicron variant, especially the BQ and XBB subvariants, have been rendered ineffective and are no longer FDA-authorized for treatment, pre-exposure, or post-exposure prevention of COVID-19 [100–104].

The updated guidelines (NIH, IDSA, and NICE) recommend against the use of anti-SARS-CoV-2 neutralizing monoclonal antibodies for the treatment or post-exposure prophylaxis against COVID-19. Only the NICE guideline still recommends sotrovimab as an option for the treatment of COVID-19 in adult and pediatric (≥ 12 years) patients with an increased risk of disease progression only if Paxlovid treatment is not applicable [38–40].

#### Convalescent plasma (CP)

For years, the CP has been used for the treatment of many severe acute viral infections, such as SARS, MERS, and influenza outbreaks, and recently in the treatment of COVID-19 [105]. CP is collected from patients who have recovered from a viral infection to transfuse virus-neutralizing antibodies (Abs) to give the recipient a sort of passive immunity [106]. The main components of CP are neutralizing antibodies (IgM and IgG), clotting factors, anti-inflammatory cytokines, protein C, and protein S, which help to ameliorate the infection [107].

Anti-SARS-CoV-2 neutralizing Abs in CP might have multiple potential mechanisms of action in COVID-19. The Abs are directed against the RBD of the S protein to interfere with its interaction with the ACE2 receptor, thus preventing viral entry into the host cell. Abs in CP also inhibit the complement factors C3a and C5a and decrease immune complex formation [108]. The transfused IgG in CP can also neutralize cytokines such as IL-1β and TNFα and limit the inflammatory response triggered by excessive complement activation. Additionally, CP is found to enhance dendritic cell anti-inflammatory functions, which could be important in cases of excessive inflammation due to infection [109].

The NIH and the IDSA guidelines recommend against the use of CP for the treatment of COVID-19 in hospitalized patients, especially if this CP was collected “prior to the emergence of the Omicron (B.1.1.529) variant” [38, 39].

Adverse effects of CP are transfusion reactions such as allergic reactions, anaphylactic reactions, febrile nonhemolytic reactions (< 1% of all transfusions) [39], transfusion-associated circulatory overload, transfusion-related acute lung injury, transfusion-transmitted infections (e.g., HIV, hepatitis B, hepatitis C), hypothermia, metabolic complications, and post-transfusion purpura [110–112].

#### SARS-CoV-2 specific immunoglobulins

Intravenous immunoglobulin (IVIg) is used as an adjunctive treatment for many diseases, including, but not limited to, Guillain–Barre syndrome, myasthenia gravis, immune-cytopenias, vasculitis, SLE, and Kawasaki syndrome. Also, they have been used in the treatment of some infections, such as the Parvovirus B19 infection [113]. A systematic review of four clinical trials and three cohort studies concluded that the use of IVIg in the critical subgroup (ARDS, sepsis, septic shock requiring MV) could decrease mortality compared to the control group, but no significant differences were reported in the severe (respiratory rate > 30 BPM, PaO2/FiO2 ≤ 300 mmHg) or non-severe subgroups [114].

Currently, no sufficient clinical data is available on the use of these agents in COVID-19. The NIH recommends against using SARS-CoV-2-specific immunoglobulin for the treatment of patients with acute COVID-19. Potential risks may include transfusion reactions. Theoretical risks may include antibody-dependent enhancement of infection [38].

### Anti-inflammatory drugs and immunomodulators:

COVID-19 is characterized by an exacerbated inflammatory response with an increased incidence of a cytokine storm which represents the major mechanism of organ damage in COVID-19. Hence, anti-inflammatory/immunomodulator drugs may be of immense importance in the management of COVID-19-associated inflammatory damage. In this section, we discuss the most widely used anti-inflammatory/immunomodulator drugs during the COVID-19 pandemic.

#### Corticosteroids

Patients with COVID-19 could experience a systemic inflammatory response that may cause lung injury and multi-organ dysfunction; corticosteroids, with their known effective anti-inflammatory action, are thought to prevent these outcomes. Favourable effects were reported with the use of corticosteroids in patients with lung infections like Pneumocystis jirovecii pneumonia with hypoxemia [115]. Using corticosteroids in patients with ARDS accelerated clinical improvement and reduced mortality rates [116, 117].

This class is used for the control of many auto-immune diseases and to maintain graft survival after organ transplantation due to their strong anti-inflammatory properties. They were found to be beneficial with COVID-19, especially in hospitalized patients who required O_2_ therapy, mostly due to their role in ameliorating the COVID-19-induced systemic inflammation [118].

The NIH, the IDSA, and the NICE guidelines recommend the use of dexamethasone in “hospitalized patients with severe COVID-19” [38–40]. The recommended dose of dexamethasone is 6 mg IV or PO for 10 days or until discharge. If it is not available, an alternative corticosteroid with an equivalent dose may be used, such as prednisone 40 mg, methylprednisolone 32 mg, or hydrocortisone 160 mg, which are used in the management of shock in COVID-19 patients (as dexamethasone lacks mineralocorticoid activity, which renders it less effective for sodium and fluid retention) [119]. The pediatric dose of dexamethasone is 0.15 mg/kg/dose (maximum dose: 6 mg) once daily for up to 10 days [38].

Patients receiving a short course of steroids may experience hyperglycemia, neuropsychiatric symptoms, an increased risk of opportunistic fungal infections (e.g., mucormycosis, aspergillosis), and reactivation of latent infections (e.g., HBV, herpesvirus infections, tuberculosis). Patients who are receiving inhaled corticosteroids may develop oral candidiasis [119]. During corticosteroid treatment, we should monitor patients (especially if taken with other immunosuppressant drugs) for adverse effects with systemic forms like opportunistic infections such as mucormycosis [120, 121] and dormant infections [38].

Mucormycosis, or the deadly black fungus, is a life-threatening fungal infection caused by mucormycetes. It has been associated with conditions where low immunity takes place, such as in the case of diabetes, neutropenia, organ transplantation, burns, hematological malignancies, steroid use, IV drug usage, renal disease, and the use of broad-spectrum antibiotics. It is becoming common among COVID-19 patients, where factors such as high body temperature, high osmolarity, and hypoxia are present. Moreover, wearing O_2_ masks or being on a ventilator could provide an entry path to the body for the fungus [122].

Treatment of mucormycosis associated with COVID-19 does not differ from non-COVID patients. Treatment options include early and aggressive surgical resection and debridement of the affected tissues. The drug of choice for first-line therapy of mucormycosis is liposomal amphotericin B. It needs to be initiated early and is strongly recommended at a dose of 5 mg/kg per day in 200 ml of 5% dextrose over 2–3 h for 3–6 weeks [123]. Other antifungals, such as posaconazole or isavuconazole, have also been described for the treatment of mucormycosis associated with COVID-19 [124].

#### Interleukin-6 inhibitors: Tocilizumab and Sarilumab

Interleukin-6 is a pro-inflammatory cytokine released by inflammatory cells such as lymphocytes and monocytes and is found to be produced in excessive amounts by the epithelial cells during SARS-CoV infection [125].

It is thought that by modulating the extent of IL-6 activity, the course of COVID-19 illness, duration, and severity could be modified. Tocilizumab and sarilumab are humanized anti-interleukin-6 receptor mAbs that are thought to exhibit potent anti-inflammatory effects with improvements in morbidity and mortality in patients with COVID-19 [38].

In December 2022, tocilizumab was FDA-approved for the treatment of COVID-19 in hospitalized adults who require supplemental O_2_, mechanical ventilation, or ECMO [126]. NIH, IDSA, and NICE guidelines recommend the use of tocilizumab in addition to dexamethasone for the treatment of hospitalized patients “with progressive severe or critical COVID-19 who have elevated markers of systemic inflammation”. Sarilumab could be used if tocilizumab could not be used [38–40].

Indeed, multiple trials demonstrated that tocilizumab treatment did not exhibit clinical improvement in patients with COVID-19-associated pneumonia, with concerns regarding its safety [127–129]. On the other side, multiple studies demonstrated that tocilizumab plus standard of care therapy was associated with a significant reduction in progression to mechanical ventilation and death [130]. In general, tocilizumab is not recommended as routine therapy for patients with COVID-19; rather, it should be considered for selected “critical’ cases [43].

Tocilizumab and sarilumab adverse effects include elevated liver enzymes (dose-dependent), infusion-related reactions, and hypersensitivity reactions [131–133]. Other adverse effects, such as a runny nose, sore throat, sinus infection, headache, and increased blood pressure, were reported. Very rarely, GIT perforations may occur [133].

These mAbs actively cross the placenta with the greatest level in the third trimester and may affect the immunity of the fetus, however, no sufficient data indicates whether they lead to abortion or major birth defects or not. Hence, currently, it is not recommended to use them during pregnancy, and there is no sufficient data for justification of their use in children [134].

Tocilizumab and sarilumab should be used cautiously in patients who are immunosuppressed or receiving immunosuppressive drugs, their ALT levels > 5 times the upper limit of normal, are at elevated risk for gastrointestinal perforation, have an uncontrolled serious infection other than COVID-19, their absolute neutrophil counts < 500 cells/µL, their platelet counts < 50,000 cells/µL, or the presence of hypersensitivity to these drugs [131, 132].

#### Interleukin-1 inhibitors: Anakinra

The endogenous IL-1 is found to be elevated in COVID-19 patients [135, 136]. Il-1β released due to respiratory epithelial damage leads to the recruitment of inflammatory cells with more generation of pro-inflammatory cytokines. IL-1 receptor blockers such as “anakinra” or drugs that block IL-1 signalling like “canakinumab” can interrupt this cycle and are under investigation for COVID-19 [137].

Anakinra is a recombinant human IL-1 receptor antagonist. It is FDA-approved for the treatment of rheumatoid arthritis and cryopyrin-associated periodic syndromes [138]. The NIH does not recommend for or against its use in COVID-19 due to the limited clinical evidence [38]. The IDSA guideline suggests against its routine use in hospitalized patients with severe COVID-19 [39]. In November 2022, anakinra was FDA-authorized for the treatment of COVID-19 in hospitalized adults with pneumonia requiring supplemental O_2_ who are at high risk of disease progression and have an elevated plasma soluble urokinase plasminogen activator receptor (suPAR) [139].

With anakinra, there is an increased risk of infection reported if this drug is used with TNF-α blockers for a prolonged time, but not with short-term use. Headache, nausea, vomiting, and elevation of liver enzymes are commonly reported side effects of anakinra [140]. The American College of Rheumatology (ACR) recommends against the use of anakinra during pregnancy [141].

Canakinumab is a human monoclonal antibody against the beta subunit of IL-1. It is FDA-approved for the treatment of systemic juvenile idiopathic arthritis and Still’s disease. Its common adverse effects are hypersensitivity reactions, neutropenia, nasopharyngitis, headache, abdominal pain, nausea, vomiting, diarrhea, musculoskeletal pain, injection site reactions, and elevation of liver enzymes with an increased risk of infections, including respiratory tract infections, bronchitis, gastroenteritis, and pharyngitis [142]. Due to the lack of clinical evidence, the NIH recommends against the use of this agent for the treatment of COVID-19 [38].

#### Janus kinase (JAK) inhibitors

Cytokines play key roles in controlling cell functions like cell growth, survival, and immune response. They work by activating specific cytokine receptors that rely on the Janus kinase family in their signal transduction. Janus kinase acts through the phosphorylation of activated cytokine receptors, which in turn activate the signal transducer and activator of transcription (STAT) proteins, which modulate gene transcription [143]. Accordingly, inhibiting Janus kinase activity will lead to the blockade of cytokine signalling, thus decreasing the immune response in many diseases, such as rheumatoid arthritis [144]. As COVID-19 is characterized by a cytokine storm, the use of Janus kinase inhibitors may play a role in decreasing such a hyperinflammation state to achieve clinical improvement for COVID-19 patients.

##### Baricitinib

Baricitinib is FDA-approved for the treatment of rheumatoid arthritis [145]. It acts through inhibition of JAK1/JAK2, thus inhibiting the inflammatory cascade; it also shows inhibition of IL-6-induced STAT3 phosphorylation. Additionally, it has a direct antiviral effect through inhibition of viral entry into the host cell [146].

In May 2022, baricitinib was FDA-approved for the treatment of COVID-19 in hospitalized adults requiring supplemental O_2_, mechanical ventilation, or ECMO [147]. The NIH, IDSA, and NICE guidelines recommend the use of baricitinib in addition to dexamethasone (or remdesivir) for the treatment of hospitalized adult and pediatric (≥ 2 years) patients with severe COVID-19 [38–40].

Adverse effects of baricitinib may include hypersensitivity reactions, infections such as respiratory and urinary tract infections, reactivation of herps, myelosuppression, thrombosis, elevation of liver enzymes, GIT perforation (in rare cases), and serious cardiac-related events (myocardial infarction and stroke). Baricitinib needs dose adjustment in renal patients. It is a CYP-3A4 substrate with drug interactions with CYP-3A4 inducers and inhibitors [146].

##### Tofacitinib

Tofacitinib is another JAK inhibitor approved for the treatment of rheumatoid arthritis, psoriatic arthritis, and ulcerative colitis [148]. Its use was associated with serious adverse reactions, including cardiovascular events, stroke, and death [149]. It is also a CYP-3A4 substrate, so the dose should be monitored in cases where it is co-administered with CYP-3A4 inhibitors, and it is not recommended to be used with CYP-3A4 strong inducers [149]. A complete blood count and liver and kidney functions should be requested before initiating JAK inhibitors. Screening for viral hepatitis and tuberculosis is recommended [150].

The NIH guideline states that oral tofacitinib could be used instead of oral baricitinib if baricitinib therapy is not applicable [38]. The IDSA guideline suggests tofacitinib for hospitalized adults with severe COVID-19, not on mechanical ventilation [39].

#### Complement component “C5a” inhibitor: Vilobelimab

The activation of complement pathways is thought to play pivotal roles in immune activation and cellular damage manifested in COVID-19. The complement component “C5a” is a potent anaphylatoxin that attracts neutrophils, macrophages, and monocytes to the site of infection, which triggers tissue damage via oxidative radical formation, histamine release, and exaggerated cytokine release [151]. Vilobelimab is a monoclonal antibody against C5a that is thought to reduce immune system activation through inhibition of lung injury [38].

In April 2023, vilobelimab was FDA-authorized for the treatment of hospitalized adults with severe COVID-19 when initiated within 48 h of receiving invasive mechanical ventilation, or ECMO [152]. Due to insufficient evidence, the NIH guideline does not recommend either for or against the use of vilobelimab for the treatment of COVID-19 [38].

The commonest adverse effects of vilobelimab included pneumonia, sepsis, and infections such as herpes simplex, enterococcal infection, and bronchopulmonary aspergillosis, in addition to pulmonary embolism, deep venous thrombosis, hypertension, thrombocytopenia, elevated liver enzymes, and rash [38].

#### Currently used, not approved COVID-19 anti-inflammatory drugs: Non-steroidal anti-inflammatory drugs (NSAIDS)

NSAIDs were often used in the early stages of the COVID-19 pandemic to treat fever, body aches, and headaches, which are frequently encountered symptoms in COVID-19 patients [153]. However, at that time, some reports suggested that the use of NSAIDs was linked to worsened infection severity and poorer clinical outcomes, which was postulated to be due to the upregulation of angiotensin-converting enzyme (ACE) 2 expression, which may facilitate viral host cell invasion [23, 154]. Over time, and with the emergence of many well-designed studies, it was revealed that NSAIDs do not influence the expression of this enzyme [155], and it was shown that there is no evidence supporting these assertions; this is reflected in the current recommendations from the major authorities across the world, which encourage the use of NSAIDs as analgesics and antipyretics during COVID-19 [153]. Many studies reported favorable effects of ibuprofen in attenuation of symptoms, reduction of hospital length, incidence of ICU admission, and improvement of leucocytic/lymphocytic count in patients with COVID-19 [156, 157].

#### Obsolete COVID-19 anti-inflammatory drugs: Colchicine

Colchicine is an anti-inflammatory drug used in diseases like gout, pericarditis, and familial Mediterranean fever (FMF) [158]. It was also shown to reduce cardiovascular events in patients with coronary artery disease [159]. Colchicine disrupts microtubule assembly, thus inhibiting neutrophil chemotaxis. It also inhibits inflammasome signalling and decreases cytokine formation like IL-6 and IL-1β [160]. Having these anti-inflammatory properties added to its relative safety and limited immunosuppressive effects favored the use of colchicine in the early times of the COVID-19 pandemic. However, at the current time, the NIH, IDSA, and NICE guidelines recommend against the use of this agent for the treatment of COVID-19 [38–40].

Colchicine has side effects like nausea, vomiting, diarrhea, and abdominal cramping, but, in rare cases, it may cause neurotoxicity, myopathy, and bone marrow depression. It should be avoided in patients with severe renal insufficiency, and it should be monitored in patients with moderate renal insufficiency. Colchicine should be cautiously used with other drugs that are CYP-3A4 or P-glycoprotein inhibitors, as such interaction will increase the level of colchicine in plasma, raising the risk of adverse effects. There is an increased risk of myopathy if co-administrated with statins due to competition on the CYP-3A4 and P-glycoprotein pathways [160, 161].

It crosses the placenta, and due to its anti-mitotic effect, it was thought to have a teratogenic effect; however, a meta-analysis concluded that the use of colchicine during pregnancy did not cause major fetal malformations [162].

## Conclusions

Over the past 3 years, the COVID-19 pandemic has continued to strain healthcare systems, posing a significant threat to public health worldwide. The SARS-CoV-2 pandemic has led to over 6 million deaths as of October 2023. There is a wide diversity of therapeutic strategies that have been used for the treatment of COVID-19 at various stages of the disease, including antivirals, anti-SARS-CoV-2 antibody agents (monoclonal antibodies, convalescent plasma, and immunoglobulins), anti-inflammatory drugs, and immunomodulators. Nevertheless, one of the most prominent therapeutic challenges in producing effective anti-COVID drugs is SARS-CoV-2 mutations and emerging new variants and subvariants. Currently, the anti-COVID-19 drug pipeline continuously affords novel treatments to face these medication challenges.

### Supplementary Information

**Additional file 1. Additional Table 1:** Updates of COVID-19 treatment guideline recommendations (NIH, IDSA, and NICE) and FDA approval/EUA for drug therapy of COVID-19.
