# Supplementary material for: What are the current anti-COVID-19 drugs? From traditional to smart molecular mechanisms
Source: Virol J. 2023 Oct 24;20:241. doi: 10.1186/s12985-023-02210-z (PMC10594888; doi:10.1186/s12985-023-02210-z)
Supplement: Supplementary file 1 — Additional file 1. Additional Table 1: Updates of COVID-19 treatment guideline recommendations (NIH, IDSA, and NICE) and FDA approval/EUA for drug therapy of COVID-19. [file 12985_2023_2210_MOESM1_ESM.docx]

**Table 2: Updates of COVID-19 treatment guideline recommendations (NIH, IDSA, and NICE) and FDA approval/EUA for drug therapy of COVID-19.**

| **Drug** | **NIH Guidelines** [39] | | **IDSA Guidelines** [40] | | **NICE Guidelines** [41] | | **FDA Approval/ EUA** |
| --- | --- | --- | --- | --- | --- | --- | --- |
| 1. **Antiviral Drugs** | | | | | | | |
| **1.** **Remdesivir** | - NIH recommends the use of remdesivir for non-hospitalized patients ≥ 12 years at high risk of progressing to severe COVID-19.  - NIH recommends the use of remdesivir in hospitalized patients ≥ 12 years who don’t require supplemental O_2_ but are at high risk of progressing to severe COVID-19.  - NIH recommends the use of remdesivir with or without dexamethasone in hospitalized patients ≥ 12 years requiring conventional O_2_.  - NIH recommends adding remdesivir to dexamethasone + (baricitinib or tocilizumab) in hospitalized patients ≥ 12 years requiring O_2_ through HFNC or NIV. | | *- “Among patients (ambulatory or hospitalized) with mild-to moderate COVID-19 at high risk for progression to severe disease, the IDSA guideline panel suggests remdesivir initiated within seven days of symptom onset rather than no remdesivir”.*  *- “In patients on supplemental oxygen but not on mechanical ventilation or ECMO, the IDSA panel suggests treatment with five days of remdesivir rather than 10 days of remdesivir”.*  *- “In hospitalized patients with severe COVID-19, the IDSA panel suggests remdesivir over no antiviral treatment”.*  *- “In patients with COVID-19 on invasive ventilation and/or ECMO, the IDSA panel suggests against the routine initiation of remdesivir”.* | | *- “Consider a 3-day course of remdesivir for children and young people who weigh at least 40 kg and adults with COVID-19 who do not need supplemental oxygen for COVID-19, and*  *are within 7 days of symptom onset, and*  *are thought to be at high risk of progression to severe COVID-19”.*  *- “Consider a course of remdesivir (up to 5 days) for people who have COVID-19 pneumonia and are in hospital and need low-flow supplemental oxygen”.*  *- “Do not use remdesivir for COVID-19 pneumonia in anyone in hospital and on high-flow nasal oxygen, continuous positive airway pressure, non-invasive mechanical ventilation or invasive mechanical ventilation, except as part of an ongoing clinical trial”.* | | - In October 2020, remdesivir was FDA- approved for the treatment of COVID-19 in hospitalized adults and pediatric patients (aged ≥12 years and weighing ≥ 40 kg).  - In January 2022, the FDA expanded the approval to include the treatment of COVID-19 in non-hospitalized adults and pediatric patients (aged ≥12 years and weighing ≥ 40 kg) with mild-to-moderate COVID-19 who are at high risk for disease progression.  -In April 2022, the FDA expanded the approval to include the treatment of COVID-19 in hospitalized or non-hospitalized pediatric patients (≥ 28 days of age and weighing at least 3 Kg) with mild-to-moderate COVID-19 who are at high risk for disease progression. |
| **2. Ritonavir/**  **Nirmatrelvir (Paxlovid)** | - NIH recommends the use of Paxlovid for non-hospitalized patients ≥ 12 years at high risk of progressing to severe COVID-19. | | - *“In ambulatory patients with mild-to-moderate COVID-19 at high risk for progression to severe disease, the IDSA guideline panel suggests nirmatrelvir/ritonavir initiated within five days of symptom onset rather than no nirmatrelvir/ritonavir”.* | | - *“Nirmatrelvir plus ritonavir is recommended as an option for treating COVID-19 in adults, only if they do not need supplemental oxygen for COVID-19 and have an increased risk for progression to severe COVID-19”.* | | -In December 2021, the FDA issued an EUA for the use of Paxlovid in the treatment of mild-to-moderate COVID-19 in certain adults and pediatric patients (≥12 years of age and ≥ 40 kg) who are at high risk for progression to severe COVID-19.  - In May 2023, Paxlovid was approved for the treatment of mild-to-moderate COVID-19 in adults who are at high risk for progression to severe COVID-19, including hospitalization or death. |
| **3. Molnupiravir** | - NIH recommends the use of molnupiravir for non-hospitalized adults at high risk of progressing to severe COVID-19 as an alternative therapy to Paxlovid and remdesivir.  - NIH recommends against the use of molnupiravir for the treatment of COVID-19 in pregnant patients unless there are no other options and therapy is clearly indicated. | | ***- "In ambulatory patients (≥18 years) with mild-to-moderate COVID-19 at high risk for progression to severe disease who have no other treatment options, the IDSA guideline panel suggests molnupiravir initiated within five days of symptom onset rather than no molnupiravir”.*** | | - *“Consider a 5-day course of molnupiravir for adults with COVID-19 who do not need supplemental oxygen for COVID-19, and are within 5 days of symptom onset, and are thought to be at high risk of progression to severe COVID-19”.* - *“Do not offer molnupiravir to children and young people aged under 18, or pregnant women”.* | | -In December 2021, the FDA issued an EUA for the use of molnupiravir in the treatment of mild-to-moderate COVID-19 in adults who are at high risk for progressing to severe COVID-19 and for whom alternative COVID-19 treatment options approved or authorized by the FDA are not accessible or clinically appropriate. |
| 1. **Monoclonal antibodies (anti-SARS-CoV-2 monoclonal antibodies)** | | | | | | | |
| **- Bamlanivimab/ Etesevimab**  **- Casirivimab/**  **Imdevimab**  **- Sotrovimab**  **- Bebtelovimab**  **- Tixagevimab/ Cilgavimab** | | -NIH recommends against the use of Bamlanivimab/etesevimab or casirivimab/imdevimab for the treatment of COVID-19.  -NIH recommends against using tixagevimab/ cilgavimab as SARS-CoV-2 pre-exposure prophylaxis.  - NIH recommends against the use of anti-SARS-CoV-2 mAbs for the treatment or prevention of COVID-19 because the dominant Omicron subvariants in the United States are not expected to be susceptible to these products. | | *- Recommendation for the use of* Bamlanivimab/etesevimab *or casirivimab/imdevimab as post-exposure prophylaxis was retired.*  *- Recommendation for the use of bebtelovimab for the treatment of COVID-19 was retired.*  *- Tixagevimab/cilgavimab is no longer authorized for Pre-Exposure Prophylaxis use in the US until further notice by FDA.* | | *- “Sotrovimab is recommended as an option for treating COVID-19 in adults and young people aged 12 years and over and weighing at least 40 kg, only if: • they do not need supplemental oxygen for COVID-19 and they have an increased risk for progression to severe COVID-19, and nirmatrelvir plus ritonavir is contraindicated or unsuitable”.*  *- “Casirivimab plus imdevimab is not recommended, within its marketing authorisation, for treating COVID-19”.* | - In 2022 and January 2023, the FDA announced that Bamlanivimab/etesevimab, casirivimab/imdevimab, Sotrovimab, Bebtelovimab, and tixagevimab/cilgavimab were no longer authorized in any U.S. region due to the high frequency of circulating SARS-CoV-2 variants that are non-susceptible to them. Therefore, these drugs may not be administered for treatment, pre-exposure, or post-exposure prevention of COVID-19 under the Emergency Use Authorization until further notice by the Agency. |
| 1. **COVID-19 convalescent plasma (CCP)** | | | | | | | |
|  | - The NIH “recommends against the use of CCP for the treatment of COVID-19 in hospitalized, immunocompetent patients”. - NIH does not recommend either for or against the use of high-titer CCP for the treatment of COVID-19 in patients who are immunocompromised. | | - *“Among immunocompetent patients hospitalized with COVID-19, the IDSA guideline panel recommends against COVID-19 convalescent plasma”.* - *“Among immunocompromised patients hospitalized with COVID-19, the IDSA guideline panel suggests against the routine use of COVID-19 convalescent plasma”.* - *“Among ambulatory patients with mild-to-moderate COVID-19 at high risk for progression to severe disease who have no other treatment options, the IDSA guideline panel suggests FDA-qualified high-titer COVID-19 convalescent plasma within 8 days of symptom onset rather than no high-titer COVID-19 convalescent plasma”.* | | - Not mentioned. | | -In August 2020, the FDA issued an EUA for the use of CCP with high titers of anti-SARS-CoV-2 antibodies for the treatment of COVID-19 in patients with immunosuppressive disease or receiving immunosuppressive treatment, in inpatient or outpatient settings. |
| 1. **Anti-inflammatory and immunomodulator drugs** | | | | | | | |
| **1. Corticosteroids** | - For non-hospitalized adults with COVID-19 who are receiving dexamethasone or another corticosteroid for an underlying condition, NIH recommends continuing this therapy as directed by their health care provider.  -In non-hospitalized adults with COVID-19, NIH recommends against the use of dexamethasone or other systemic corticosteroids in the absence of another indication.  -In hospitalized adults with COVID-19 who don’t require supplemental O_2_, NIH recommends against the use of dexamethasone.  -In hospitalized adults with COVID-19 who require conventional O_2_ or O_2_ through HFNC, NIV, MV, or ECMO, NIH recommends the use of dexamethasone alone or plus baricitinib or tocilizumab.  - NIH reported that there is insufficient evidence to recommend either for or against the use of inhaled corticosteroids in hospitalized or non-hospitalized COVID-19 patients. | | *- “Among hospitalized critically ill patients with COVID-19, the IDSA guideline panel recommends dexamethasone rather than no dexamethasone”.*  *- “Among hospitalized patients with severe, but non-critical, COVID-19, the IDSA guideline panel suggests dexamethasone rather than no dexamethasone”.*  *- “Among hospitalized patients with mild-to-moderate COVID-19 without hypoxemia requiring supplemental oxygen, the IDSA guideline panel suggests against the use of glucocorticoids”.*  *- “Among ambulatory patients with mild-to-moderate COVID-19, the IDSA guideline panel suggests against inhaled corticosteroids”.* | | *- “Offer dexamethasone, or either hydrocortisone or prednisolone when dexamethasone cannot be used or is unavailable, to people with COVID-19 who need supplemental oxygen to meet their prescribed oxygen saturation levels or have a level of hypoxia that needs supplemental oxygen but who are unable to have or tolerate it. Continue corticosteroids for up to 10 days unless there is a clear indication to stop early, which includes discharge from hospital or a hospital-supervised virtual COVID ward”.*  *- “Do not use corticosteroids to treat COVID-19 in people who do not need supplemental oxygen”.*  *- “Only use budesonide to treat COVID-19 as part of a clinical trial”.* | |  |
| **2. Anti-IL-6 monoclonal antibodies:**  **- Tocilizumab**  **- Sarilumab** | -NIH recommends the use of tocilizumab added to dexamethasone and remdesivir in hospitalized adults on conventional O_2_ who have rapidly increasing O_2_ needs and systemic inflammation.  **-**NIH recommends adding tocilizumab to dexamethasone for the treatment of hospitalized adults requiring O_2_ through HFNC, NIV, MV, or ECMO.  - IV sarilumab could be used instead of IV tocilizumab if tocilizumab is not available/feasible to use. | | *- “Among hospitalized* *adults with progressive severe or critical COVID-19* *who have elevated markers of systemic inflammation, the IDSA guideline panel suggests tocilizumab in addition to standard of care (i.e., steroids) rather than standard of care alone”.*  *- “When tocilizumab is not available for patients who would otherwise qualify for tocilizumab, the IDSA guideline panel suggests sarilumab in addition to standard of care (i.e., steroids) rather than standard of care alone”.* | | *- “Tocilizumab is recommended, within its marketing authorisation, as an option for treating COVID-19 in adults who are having systemic corticosteroids and need supplemental oxygen or mechanical ventilation”.* | | -In June 2021, the FDA issued an EUA for the use of tocilizumab for the treatment of COVID-19 in hospitalized adults and pediatric patients aged ≥2 years who are receiving systemic corticosteroids and require supplemental O_2_, NIV, MV, or ECMO.  - In December 2022, tocilizumab was approved for the treatment of COVID-19 in hospitalized adults who are receiving systemic corticosteroids and require supplemental oxygen, non-invasive or invasive mechanical ventilation, or ECMO. |
| **3. IL-1 Inhibitors**  **- Anakinra**  **- Canakinumab** | - NIH does not recommend either for or against the use of anakinra for the treatment of COVID-19 due to insufficient evidence. - NIH *recommends against “the use of canakinumab* *for the treatment of COVID-19, except in a clinical trial”.* | | *- “In hospitalized patients with severe COVID-19, the IDSA guideline panel suggests against the routine use of anakinra”.* | | - Not mentioned. | | - in November 2022, anakinra was authorized for the treatment of COVID-19 in hospitalized adults with pneumonia requiring supplemental oxygen (low-or high-flow oxygen) who are at risk of progressing to severe respiratory failure and likely to have an elevated plasma soluble urokinase plasminogen activator receptor (suPAR). |
| **4. JAK Inhibitors**  **- Baricitinib**  **- Tofacitinib** | -NIH recommends the use of baricitinib added to dexamethasone and remdesivir in hospitalized adults on conventional O2 who have rapidly increasing O2 needs and systemic inflammation.  -NIH recommends adding baricitinib to dexamethasone for the treatment of hospitalized adults requiring O2 through HFNC, NIV, MV, or ECMO.  - PO tofacitinib could be used instead of PO baricitinib if baricitinib is not available/feasible to use.  - NIH recommends against the use of JAK inhibitors other than baricitinib or tofacitinib for the treatment of COVID-19, except in a clinical trial. | | *- “Among hospitalized adults with severe COVID-19, the IDSA panel suggests baricitinib with corticosteroids rather than no baricitinib”.*  *- “Among hospitalized patients with severe COVID-19 who cannot receive a corticosteroid (which is standard of care) because of a contraindication, the IDSA guideline panel suggests use of baricitinib with remdesivir rather than remdesivir alone”.*  *- “Among hospitalized adults with severe COVID-19 but not on non-invasive or invasive mechanical ventilation, the IDSA panel suggests tofacitinib rather than no tofacitinib”.* | | *- “Consider baricitinib for people 2 years and over in hospital with COVID-19 who need supplemental oxygen for COVID-19 and are having or have completed a course of corticosteroids such as dexamethasone, unless they cannot have corticosteroids, and have no evidence of infection (other than SARS-CoV-2) that might be worsened by baricitinib”.* | | - In May 2022, baricitinib was approved for the treatment of COVID-19 in hospitalized adults requiring supplemental oxygen, non-invasive or invasive mechanical ventilation, or ECMO. |
| **5. Vilobelimab** | - NIH does not recommend either for or against the use of vilobelimab for the treatment of COVID-19 due to insufficient evidence. | | - Not mentioned. | | - Not mentioned. | | In April 2023, the FDA issued an EUA for the use of vilobelimab for the treatment of COVID-19 in hospitalized adults when initiated within 48 hours of receiving invasive mechanical ventilation or ECMO. |
